# HBx induced AFP receptor expressed to activate PI3K/AKT signal to promote expression of Src in liver cells and hepatoma cells

**DOI:** 10.1186/s12885-015-1384-9

**Published:** 2015-05-06

**Authors:** Mingyue Zhu, Junli Guo, Wei Li, Hua Xia, Yan Lu, Xu Dong, Yi Chen, Xieju Xie, Shigan Fu, Mengsen Li

**Affiliations:** 1 Hainan Provincial Key Laboratory of Carcinogenesis and Intervention, Hainan Medical College, Haikou, 571199 , Hainan Province P.R. China; 2 Key Laboratory of Molecular Biology, Hainan Medical College, Haikou, 571199 P.R. China; 3 Department of Pathophysiology, Hainan Medical College, Haikou, 571199 P.R. China; 4 Department of Physiology, Hainan Medical College, Haikou, 571199 P.R. China; 5 Institution of Tumor, Hainan Medical College, Haikou, 571199 P.R. China

**Keywords:** Hepatitis B virus-x(HBx), Alpha fetoprotein(AFP)/AFP receptor, PI3K/AKT signal, Hepatoma cells

## Abstract

**Background:**

Hepatitis B virus (HBV)-X protein(HBx) is a transactivator of host several cellular genes including alpha-fetoprotein(AFP) and AFP receptor(AFPR) which contributes to HBV-associated tumor development. The expression of AFP/AFPR are correlated with hepatocellular carcinoma(HCC)-initial cells. But the role of AFP and AFPR in promoting occurrence of HBV-related HCC were still unclear.

**Methods:**

A total of 71 clinical patients’ liver specimens, normal human liver cells L-02 and HCC cell lines, PLC/PRF/5 were selected for analyzing the effects of HBx on expression of AFP, AFPR and Src. The expression of goal proteins were detected by Immunohistochemical stained and Western blotting; HBx-expressed vectors were constructed and transfected into L-02 cells, laser confocal microscopy was applied to observe expression and location of AFP, AFPR and Src in the normal liver cells and HCC cells, soft agar colony formation assay was used to observe colonies formed of the cells.

**Results:**

We confirmed HBx gives preference to promote the expression of AFP and AFPR; HBx priors to up-regulate the expression of AFPR and AFP in L-02 cells and in normal liver specimens; AFPR signal been able to stimulate Src expression. The results also indicated that phosphatidylinositol 3-kinase(PI3K) inhibitors Ly294002 and GDC0941 effectively suppress AFPR mediated up-regulation expression of Src in AFPR positive HCC lines.

**Conclusions:**

HBx priors to drive the expression of AFP and AFPR to promote expression of Src in normal liver cells and hepatoma cells; AFP and AFPR maybe play pivotal role in HBV-related hepatocarcinogenesis; Targeting AFPR is an available therapeutic strategy of HCC.

**Electronic supplementary material:**

The online version of this article (doi:10.1186/s12885-015-1384-9) contains supplementary material, which is available to authorized users.

## Background

Hepatocellular carcinoma(HCC) development closely associated with infected by hepatitis B virus(HBV). HBV-X protein(HBx), a small regulatory protein of HBV that has been require for contributing to the onset and progression of HBV-related HCC [1-3]. However, the molecular mechanisms involved in HBx-mediated hepatocarcinogenesis remain to be fully elucidated. HBx emerged transcriptional activity on a variety of viral and cellular promoters [4-6]. HBx does not directly bind to genomic DNA of host cells, but has been shown to interact with components of basal transcription machinery [7,8] and several transcription factors, such as p53, HIF-1α and E4F1 [9-11]. However, documents evidenced that the localization of HBx predominantly in the cytoplasm, HBx harbors a function to activate signal transduction cascades, including phosphatidylinositol 3-kinase(PI3K)/protein kinase B(AKT) [12,13] and mitogen-activated protein kinase(MAPK) [14]. Activation of these signal pathways may contribute to HBx-mediated effects on driving malignant transformation of liver cells.

Alpha fetoprotein(AFP) is an early biomarker of HCC diagnosis, promote tumor cells proliferation effects of AFP have been reported by several groups [15,16], furthermore, data indicated that AFP play pivotal role in the hepatocarcinogenesis [17]. Our investigations found that these effects of AFP maybe mediated by AFP receptors(AFPR) [18,19], cytoplasmic AFP activated PI3K/AKT signal pathway to promote expression of some oncogenes and proliferation of HCC cells [16,20,21]. HBx priors to induce expression of AFP and AFPR to activate PI3K/AKT signal pathway in normal liver tissues and cell lines [22]. Because HBx was a critical factor for HBV driving development of HCC, HBx activates Wnt/β-catenin and Src kinase led to malignant transformation of liver cells [23], and Src plays important role in HCC development [24]. In this study, we discovered that HBx priors to induce expression of AFP and AFPR in normal liver tissues and liver cell lines via activating PI3K/AKT signal, and AFP promoted expression of Src was mediated by AFPR, AFPR signal possess a character to activate PI3K/AKT. Our results supported that AFP and AFPR as potential stimulated factors for HBx inducing hepatocarcinogenesis.

## Methods

### Clinical specimens collected

Archived clinical specimens were originally collected during hepatectomy of 71 patients at Hainan Provincial People’s Hospital between October 2008 and September 2014. Of the 71 patients, 49 were male and 22 were female. The ages ranged between 22–76 years with an average age of 49.8 years. All enrolled patients were treated with radical surgery and received no other treatments. HBV infection was diagnosed by a test of serum hepatitis B surface antigen, and circulating AFP plasma level was measured by enzyme-linked immunosorbent assay. Clinical data were obtained by retrospective chart review. Follow-up was available for all patients. A section of liver tissue about 2.0 × 2.0 × 2.0 cm was obtained from each patient immediately after the surgery. About 1.0 × 1.0 × 1.0 cm tissue samples were fixed in 10% formalin, embedded in paraffin, and routinely stained with hematoxylin and eosin. Specimens were assessed blindly and independently by two pathologists. In case of interobserver disagreement, final decisions were achieved by general consensus. All selected patients were diagnosed by histopathologic evaluation. About 1.0 × 1.0 × 1.0 cm tissue specimens were stored in formalin and liquid nitrogen. The study protocol was approved by the Ethical Committee of Hainan Provincial Peoples’ Hospital and the Science Investigation Ethical Committee of Hainan Medical College. Written informed consent was obtained from all participants.

### Immunohistochemical stained

All of clinical patients’ liver tissues were performed by immunohistochemical staining. Following deparaffinization and antigen retrieval, the slides were blocked with 3% hydrogen peroxide for 10 minutes and then incubated with mouse anti-AFP, AFP receptor(AFPR), or Src-directed antibodies (Abcam Biotech Company, Cambridge, UK) at 4°C overnight. After washing, sections were incubated with secondary goat anti-mouse antibodies (Merck-Calbiochem) at room temperature for 60 minutes and then developed with 3,3-diaminobenzidine chromogen solution in 3,3-diaminobenzidine buffer substrate(Merck Chemicals). Sections were visualized with 3,3-diaminobenzidine and counterstained with hematoxylin. All sections were visualized by microscope(Olympus).

### Cell lines

Human normal liver cell lines, L-02 cell was purchased from the Shanghai Institution of Cellular Biology, Science Academy of China and were cultured in RPMI 1640 medium supplemented with 10% fetal calf serum. The AFP-producing and HBV-infected cell line PLC/PRF/5 was gift from the Department of Cell Biology, Peking University Health Science Center and were maintained in Dulbecco’s modified Eagle’s medium(DMEM) supplemented with 10% fetal calf serum. All cell lines were cultured at 37°C in a humidified atmosphere containing 5% CO_2_.

### Generation of HBx-expressing constructs and transfects

Construction of the HBx-expressing construct (pcDNA3.1-*HBx*) and the primer used for HBx gene amplification have been previously described [22]. Lipofectamine® 2000(Beyotime Biotechological Corp, Haimen, Jaingsu, China) was used to promote pcDNA3.1-*HBx* vectors transfected into L-02 cells. Stably transfected L-02 cells were screened using G418 (Cat No. 30-234-CR, MediatechInc, Manassas, USA) and named L-02-X.

### Western blotting analysis

Western blotting was employed to assess the protein levels of AFP, AFPR and Src. Twelve clinical patients’ specimens that were randomly selected for detecting and these protein expressed in cell lines as described previously [21,22]. The cells were co-treated Ly294002 or GDC-0941(MedChem) with AFP(Sigma), and the expression of Src, pAKT(Ser473) were detected by Western blotting.

### Localization of proteins were observed by laser confocal microscopy

The staining procedure for laser confocal microscopy observing has been previously described [22]. Briefly, cells were fixed in 4% paraformaldehyde and incubated with mouse anti-human AFPR, AFP and Src antibody for 12 hours. FITC-conjugated or TRITC-conjugated secondary anti-mouse immunoglobulin G was added and incubated for 2 hours, followed by the addition of 100 μL DAPI (1 μg/mL) and 30 minutes of incubation. Cells were visualized with the Leica TCS-NT SP2 laser confocal microscopy (Leica Camera).

### Soft agar colony formation assay

Soft agar formation assays were performed to compare the clonogenic potential of L-02 and L-02-X cells in semisolid medium. Briefly, 5000 cells of L-02 or L-02-X were mixed with 0.5% soft agar and plated on a layer of 0.8% of bottom agar in 6-well plates. 2 mL of complete medium was added on the top of agar. Cells were fed twice a week, and the plates were incubated for 14 or 21 days at 37°C with 5% CO_2_. Colonies were photographed and counted with a Nikon inverted microscope(Nikon Corp., Tokyo, Japan).

### Statistical analysis

The results of multiple observations were presented as the mean ± SD of at least three separate experiments. Statistical significance was determined using x^2^ and the student’s *t* test (SPSS 11.5 software).

## Results

### Expression of AFP, AFPR and Src were stimulated during the development of HBV-related HCC

**Figure 1:**
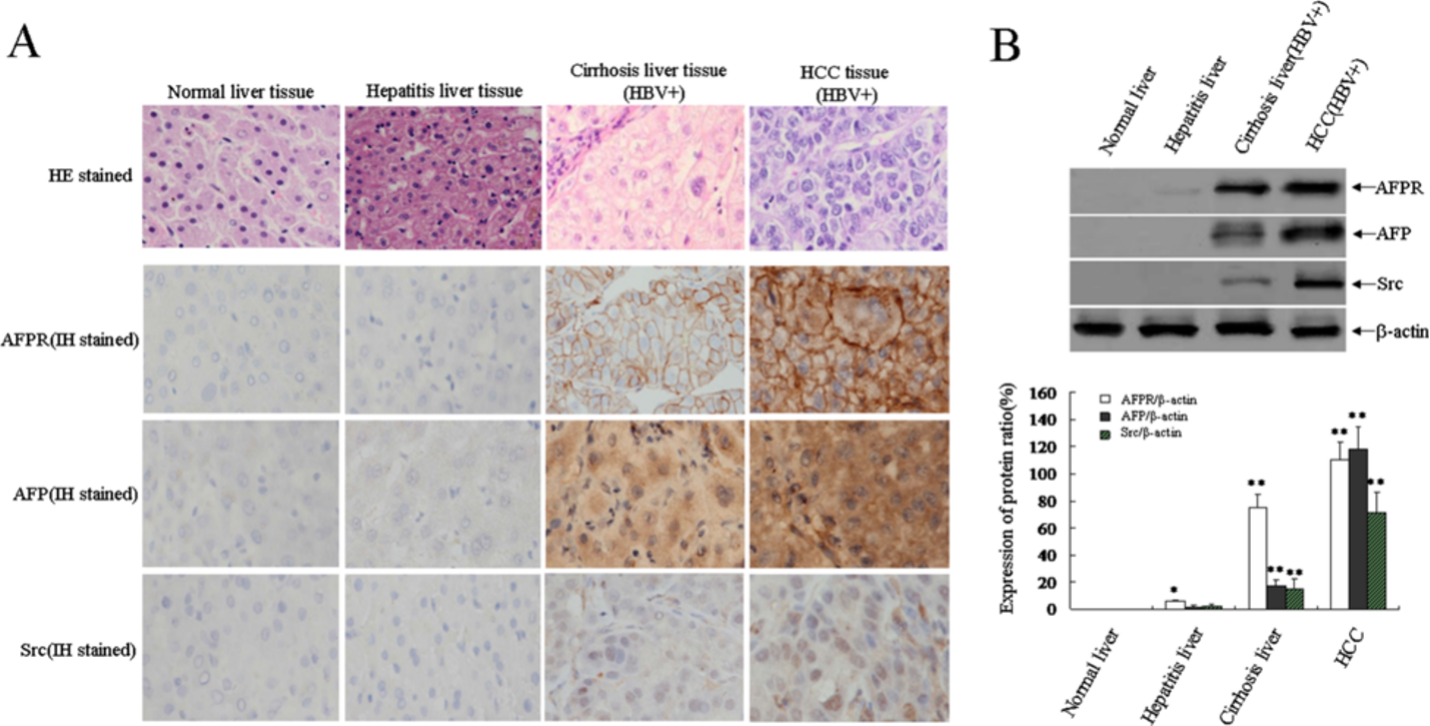
Expression of AFPR, AFP and Src in clinical patients liver tissues. **A**, Clinical liver tissues sample were collected after surgical hepatectomy, the expression of AFPR, AFP and Src in clinical patients liver tissues were detected by immunohistochemical assay. **B**, Expression of AFPR, AFP and Src in these tissues were detected by Western blotting, low column images represented the protein densitometry value contrast to internal control β-actin ratio. *P < 0.05 vs normal liver tissue groups; **P <0.01 vs normal liver tissue groups and hepatitis tissue groups. We carried out at last three reduplicate experiments. HE stained: hematoxylin-eosin stained; IH stained: immunohistochemical stained.

We studied the expression of AFP, AFPR and Src in liver tissue samples from 71 patients by immunohistochemical staining and Western blotting. The results indicated that AFP expressed in HBV-infected tissues, HBV positive cirrhosis liver tissues and HBV-related HCC tissues was 42.8%, 70.6% and 86.4% respectively; AFPR expressed in these tissues was 50.0%, 75.5% and 90.9% respectively; Src expressed in these tissues was 28.6%, 52.9% and 63.6% respectively; The levels of AFPR was significantly higher in AFP+/HBV+ liver tissues than in AFP-/HBV+ or AFP-/HBV- liver tissues (Additional file 1). Statistical analysis indicated that expression of AFP and AFPR were significantly elevated than the expression of Src during the progression of HBV-infected liver tissues to HBV-related HCC. The expression of Src also progressively elevated in HBV infected liver tissues → cirrhosis liver tissues → HBV-related HCC tissues (Additional file 1). Immunohistochemical staining indicated that AFPR located in the membrane of liver tissue cells, and much higher level in HCC tissues than in other liver tissues, expression of AFPR progressively elevation from normal liver tissue to HBV-infected tissue to cirrhotic tissue to HCC tissues (Figure 1A). Location of AFP was in cytoplasm and the location of Src both in cytoplasm and cytoblast of the cells, these protein expressed much higher level in HCC tissues than in other liver tissues (Figure 1A); Western blotting detection showed that expression of AFPR emerged in HBV-infected liver tissues, but Src and AFP expression were limited to cirrhotic and HCC tissues (Figure 1B); Moreover, the expression of AFPR and AFP significantly higher than Src in hepatitis tissues (Additional file 1 and Figure 1B). We confirmed that expression of AFPR and AFP were positively associated with liver tissues which infected with HBV and progressed of HBV-related HCC.

### HBx-expressed vectors induce expression of AFP and AFPR prior to induce expression of Src in human normal liver cells in vitro

**Figure 2:**
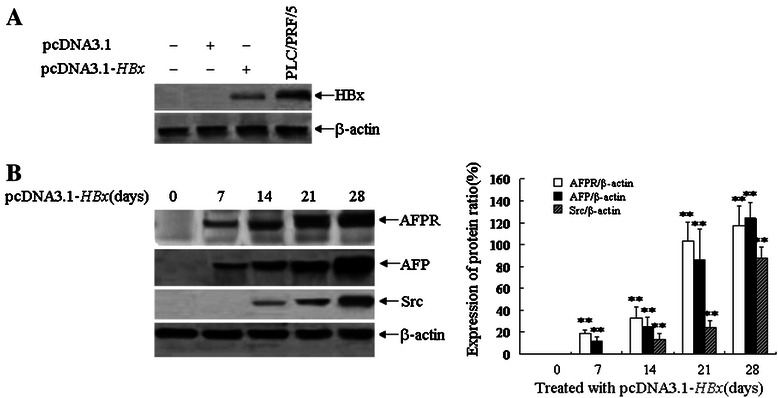
Effects of HBx on the expression of AFPR, AFP and Src in human normal liver cell line, L-02 cells. **A**, L-02 cells were transfected with pcDNA3.1-*HBx* vector for 72 hours, expression of HBx in L-02 cells were evidenced by Western blotting, human hepatoma cell line, PLC/PRF/5 cells were used as a positive control. **B**, L-02 cells were transfected with pcDNA3.1-*HBx* vectors for 0, 7, 14, 21 or 28 days respectively, expression of AFPR, AFP and Src in L-02 cells were evidenced by Western blotting, right column images represented the protein densitometry value contrast to internal control β-actin ratio. **P <0.01 vs control and 0 day treated groups. The images were representation of three reduplicate experiments.

We transfected a vector that expressing HBV protein HBx, pcDNA3.1-*HBx*, into human liver cell lines L-02 in vitro, Western blotting analysis showed that HBx expressed in L-02 cells (Figure 2A) . The pcDNA3.1-*HBx*-mediated induction of HBx expression in L-02 cells was evident at 3 days after transfection and remained elevated between 7 and 28 days after transfection (Figure 2A). In order to measure the impact of pcDNA3.1-*HBx* on the expression of AFPR, AFP and Src, L-02 cells were transfected with pcDNA3.1-*HBx* vectors and the aim proteins were detected by Western blotting. The results indicated that expression of AFPR and AFP were emerged after transfected with pcDNA3.1-*HBx* for 7 days and persisted increasing after 28 days (Figure 2B). But expression of Src start emerged after transfection for 14 days and increased after 21 days to 28 days (Figure 2B).

### Laser confocal microscopy observed confirmed that HBx promoted expression of AFPR, AFP and Src in human normal liver cell line

**Figure 3:**
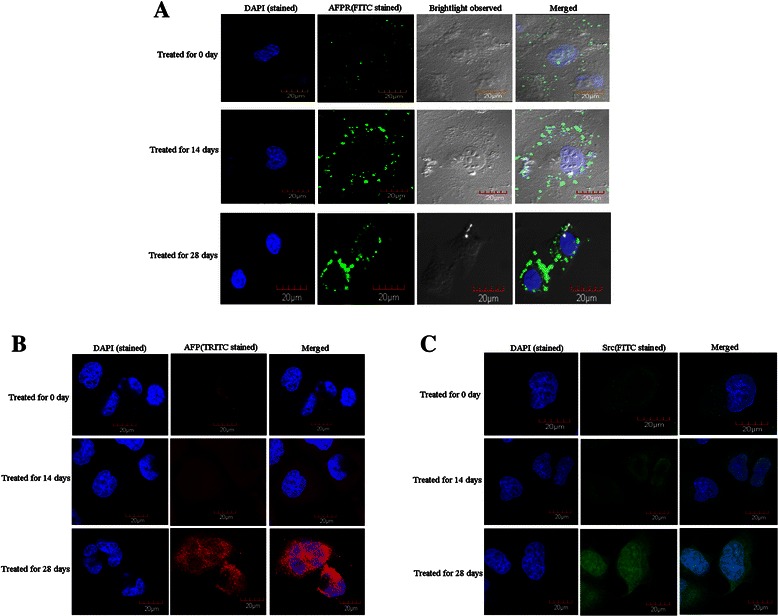
HBx induced expression of AFPR, AFP and Src in L-02 cells. L-02 cells were transfected with pcDNA3.1-*HBx* vectors for 0, 14, or 28 days respectively, expression of AFPR, AFP and Src in L-02 cells were observed by laser confocal microscopy. **A**, Expression of AFPR in L-02 cells were stained by FITC; **B**, Expression of AFP in L-02 cells were stained by TRITC; **C**, Expression of Src in L-02 cells were stained by TRITC. The images were representation of three reduplicate experiments.

While human normal liver cell line, L-02 cells were transfected with pcDNA3.1-*HBx* vectors, to evaluate the effects of pcDNA3.1-*HBx* on expression and localization of AFPR and AFP by laser confocal microscopy. The results indicated that AFPR and AFP start emerged in 14 days and significantly augmented after transfection for 28 days, AFPR located in the membrane of the cells (Figure 3A), however, AFP located in the cytoplasm of the cells (Figure 3B). Src played critical role in HBV-related HCC development [25,26]. In this investigation, furthermore, we explored the influence of HBx on the expression of Src. The results indicated that while L-02 cells were transfected with pcDNA3.1-*HBx* vectors in vitro, the expression of Src was stimulated after transfection for 14 days, and significantly elevated for 28 days. Observed by laser confocal microscopy also displayed that the location of Src not only in the cytoplasm but also in the cytoblast (Figure 3C), this appearance coincide to the location of Src in clinical HCC tissues that analyzed by immunohistochemical stained. These results confirmed that HBx been able to induce expression of AFPR, AFP and Src in human normal liver cell line. The results proved that HBx driven hepatocarcinogenesis maybe involved in promoting expression of AFPR, AFP and Src.

### AFPR signal activated PI3K/AKT to mediate AFP induced Src expression

**Figure 4:**
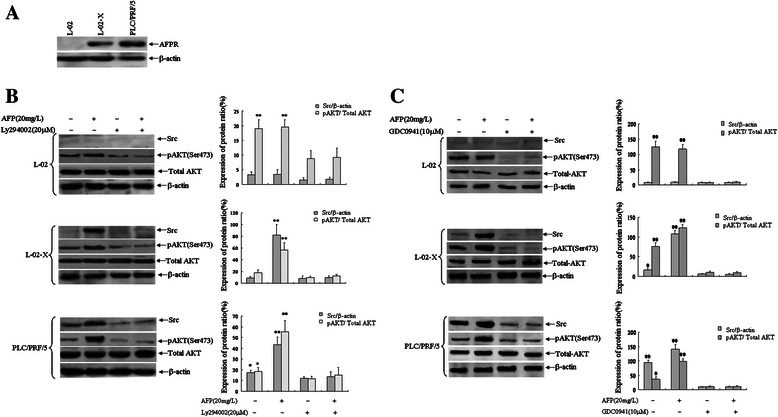
Influences of AFP on the expression of Src and pAKT(Ser473) in L-02 cells, L-02-X(stable transfected with pcDNA3.1-*HBx*) cells and PLC/PRF/5 cells. **A**, Expression of AFPR in L-02, L-02-X and PLC/PRF/5 cells were analyzed by Western blotting. **B**, L-02, L-02-X or PLC/PRF/5 cells were treated with AFP(20 mg/L), Ly294002(20 μM) or co-treated with AFP(20 mg/L) and Ly294002(20 μM) for 24 hours respectively, expression of Src and pAKT(Ser473) in these cells were analyzed by Western blotting, right column images represented the protein densitometry value compare with internal control β-actin or total AKT ratio. *P < 0.05 vs treated with AFP, Ly294002 or co-treated with AFP and Ly294002 groups; **P <0.01 vs untreated, treated with Ly294002 or co-treated with AFP and Ly294002 groups. **C**, L-02, L-02-X or PLC/PRF/5 cells were treated with AFP(20 mg/L), GDC0941(10 μM) or co-treated with AFP(20 mg/L) and GDC0941(10 μM) for 24 hours respectively, expression of Src and pAKT(Ser473) in these cells were analyzed by Western blotting, right column images represented the protein densitometry value compare with internal control β-actin or total AKT ratio. *P < 0.05 vs treated with AFP, GDC0941 or co-treated with AFP and GDC0941 groups; **P <0.01 vs untreated, treated with GDC0941 or co-treated with AFP and GDC0941 groups. The images were representation of three reduplicate experiments.

L-02 cells were stable transfected with pcDNA3.1-*HBx*, the cells were screened by G418 named L-02-X cells. Western blotting analytical results indicated that stable expression of AFPR in L-02-X cells, but AFPR could not reveal in L-02 cells. HBV positive human HCC cell line, PLC/PRF/5 also emerged expression of AFPR (Figure 4A). When L-02, L-02-X and PLC/PRF/5 cells were treated with AFP for 24 hours, the expression of Src and phosphorylated AKT(Ser473) were stimulated in L-02-X cells and PLC/PRF/5 cells, but the effects absent emerge in L-02 cells. In order to investigate whether AFPR signal been capable of activating PI3K/AKT signal pathway, PI3K specific inhibitor, Ly294002 and GDC0941 were co-treated with AFP, the results indicated that Ly294002 and GDC0941 had an antagonistic role in AFP inducing expression of Src and phosphorylated AKT(Ser473) in L-02-X cells and PLC/PRF/5 cells (Figure 4B and C). These results proved that AFPR signal was able to activate PI3K/AKT signal pathway to mediate AFP induced expression of Src in L-02-X cells and hepatoma PLC/PRF/5 cells.

### Colony formation assay confirmed that HBx induced expression of AFPR, AFP and Src to promote malignant phenotype of liver cells

**Figure 5:**
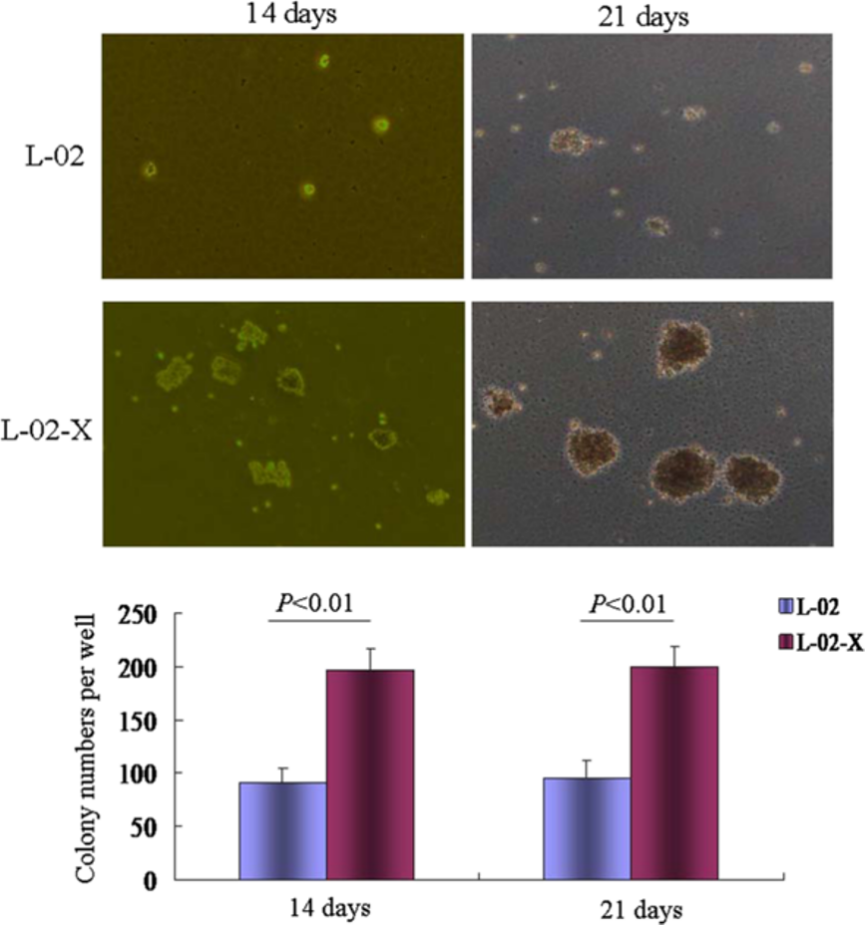
Overexpression of HBx affects colony formation of L-02 and L-02-X in soft agar. 5×10^3^/ml L-02 or L-02-X cells were cultured in soft agar for 14 or 21 days, colony formation of the cells were observed by light microscopy(×40); The low column image indicated the statistical difference of the cellular clone numbers. The images were a representation of three independent experimentations.

Expression of Src involve in the tumorigenesis. In the present study, we have found that HBx induced expression of AFPR and AFP prior to induce expression of Src in human normal liver cell line L-02. L-02 cells and stable expression HBx cell line, L-02-X cells were cultured in soft agar. The results indicated that L-02 or L-02-X cells colonigenesis in the soft agar. The number of colonies from 6 randomly selected microscopic fields was counted and an average colony number for each group was shown in Figure 5. L-02-X cells colonies number and growth significantly more than L-02 cells. Overall, these results suggest that HBx promoted expression of AFP, AFPR and Src was able to cause a more malignant phenotype in L-02 cells.

## Discussions

HBx proteins play important roles in the development of HBV-related HCC through the activation of growth signal pathways and the inactivation of tumor suppressive pathways, such as transcripted activity of p53 [9,27]. In this study, we found that overexpression of AFP, AFPR and Src in cirrhosis and HBV-related HCC tissue samples, and showed that expression of AFPR and AFP prior to expression of Src during the progress of HBV-related hepatocarcinogenesis. These results implied that HBx is involved in AFPR and AFP overexpression during HBV infection. HBx inactivation transcripted activity of p53 to alleviate p53 mediated repression of AFP expression [9], HBx also stimulated expression of Src via activating PI3K signal pathway [28], whereas, in this study, we despite found that HBx upregulated expression of AFPR, but the role played of HBx in regulating expression of AFPR is still unclear. Recently, we found that HBx promoted AFPR expression maybe involve in activating PI3K/AKT signal [22]. These results clued to that activation of PI3K/AKT signal was critical procedure for HBx driving malignant transformation of liver cells.

Recently, documents reported that AFP played pivotal role in HCC development and malignant behavior of liver cells [29-31], cytoplasmic AFP activated PI3K/AKT signal to stimulate expression of Ras, CXCR4 and Src through inhibiting activity of PTEN [16,21], AFP also inhibited the PI3K/AKT pathway through promoting ubiquitination of PTEN to stimulated malignant phenotype of HCC cells [32]. HBV infection caused malignant transformation of liver, during this course, *AFP* gene was activated in liver cells, so AFP was used as a tumor marker for early warning origination of HCC in clinical diagnosis. Previously, we have found that AFP enhanced proliferation of HCC cells was mediated by AFPR, AFPR was identified as G-protein combined receptor, AFPR signal mediated cAMP and [Ca^2+^]i transduction of receptor signal to promoted expression of N-Ras and c-myc [19], these results implied that AFPR signal was also a critical factor for HCC development. In this investigation, the results indicated that human normal liver cells, L-02 were transfected with HBx-expressed vectors been able to stimulate expression of AFPR priors to the expression of AFP and Src, the results implicated that AFP played important role in inducing malignant transformation of liver cells and enhancing HCC cells malignant behavior was mediated by AFPR.

Activation of Src foreshowed the occurrence of cancer. HBx induced HCC development involve in activation of Src [33,34]. In the present study, clinical data displayed that during progression of HCC, elevated expression of Src in liver cells were closely associated with infection of HBV, these results implied that HBx stimulated expression of Src plays an important role in HBV promoting development of HCC. HBx enhanced proliferation and anti-apoptosis and autophagy through activating transduction of PI3K/AKT signal pathway [13,35], and PTEN specific suppression of HBx-mediated cell survival through inhibiting PI3K pathway in human normal liver cells, Chang liver [36]. In this investigation, our results indicated that HBx induced expression of AFPR and location in membrane of L-02-X cells, the expression of AFPR also existed in HBV positive human liver cancer cells PLC/PRF/5. While these cells were treated with AFP, the expression of Src was stimulated, and PI3K specific inhibitor Ly294002 and GDC0941 were capable of withhold the role of AFP. These effects implicated that AFP promoted expression of Src and phosphorylation of AKT(Ser473) in AFPR positive cell lines was mediated by AFPR, it also proved that AFPR possessed a characteristic to activate transduction of PI3K/AKT signal pathway. The present study is the first time that discovered AFPR signal activated PI3K/AKT through signal cross-talk or AFPR maybe plays a role model of tyrosine protein kinase receptor. Recently, we found that HBx driven expression of AFP to activate transduction of PI3K/mTOR signal, stimulated expression of Src and CXCR4 in human normal liver cells [37], AFP also played role in promoting migration of HCC cells [38]. In this investigation, soft agar cultured experiment demonstrated that HBx induced expression of Src in normal liver cell line was able to promote colonigenesis and growth in soft agar, implied HBx driven malignant phenotype of liver cells involve in promoting expression of Src. Notwithstanding, our previously study results have confirmed that cytoplasmic AFP promoted proliferation and anti-apoptosis of HCC cells through activating growth signal and inhibiting apoptotic signal [21,39,40], but in this study, we found that HBx induced expression of AFPR and AFP to promote expression of Src in normal liver cells, activation of AFPR signal was a critical factor for HBx driving HCC occurrence. AFPR maybe is applied as a novel bio-target for therapeutics of HCC.

## Conclusions

Taken together, our findings are the first time to report HBx induced expression of AFPR, and AFPR signal possessed an identity to activate PI3K/AKT signal to promote expression of Src. AFPR plays important role in HBx driving hepatocarcinogenesis. Targeting AFPR to suppress PI3K/AKT signal is an available strategy of HCC therapeutic.

## Additional file

Additional file 1:
**Expression of AFP, AFPR and Src in clinical patients’ liver tissues.**

## Abbreviations

AFP: Alpha-fetoprotein
AFPR: AFP receptor
DMEM: Dulbecco’s Modified Eagle Medium
FCS: Fetal calf serum
PI3K: Phosphatidylinositol 3-kinase
AKT: Protein kinase B
pAKT: Phosphorylated AKT(Ser473)
PTEN: Phosphatase and tensin homolog deleted on chromosome 10
CXCR4: CXC motif chemokine receptor 4
FITC: Fluorescein-5-isothiocyanate
TRITC: Tetramethylrhodamine isothiocyanate
