# Supplementary material for: HBx induced AFP receptor expressed to activate PI3K/AKT signal to promote expression of Src in liver cells and hepatoma cells
Source: BMC Cancer. 2015 May 6;15:362. doi: 10.1186/s12885-015-1384-9 (PMC4427932; doi:10.1186/s12885-015-1384-9)
Supplement: Additional file 1: — Expression of AFP, AFPR and Src in clinical patients’ liver tissues. [file 12885_2015_1384_MOESM1_ESM.ppt]

## Slide 1
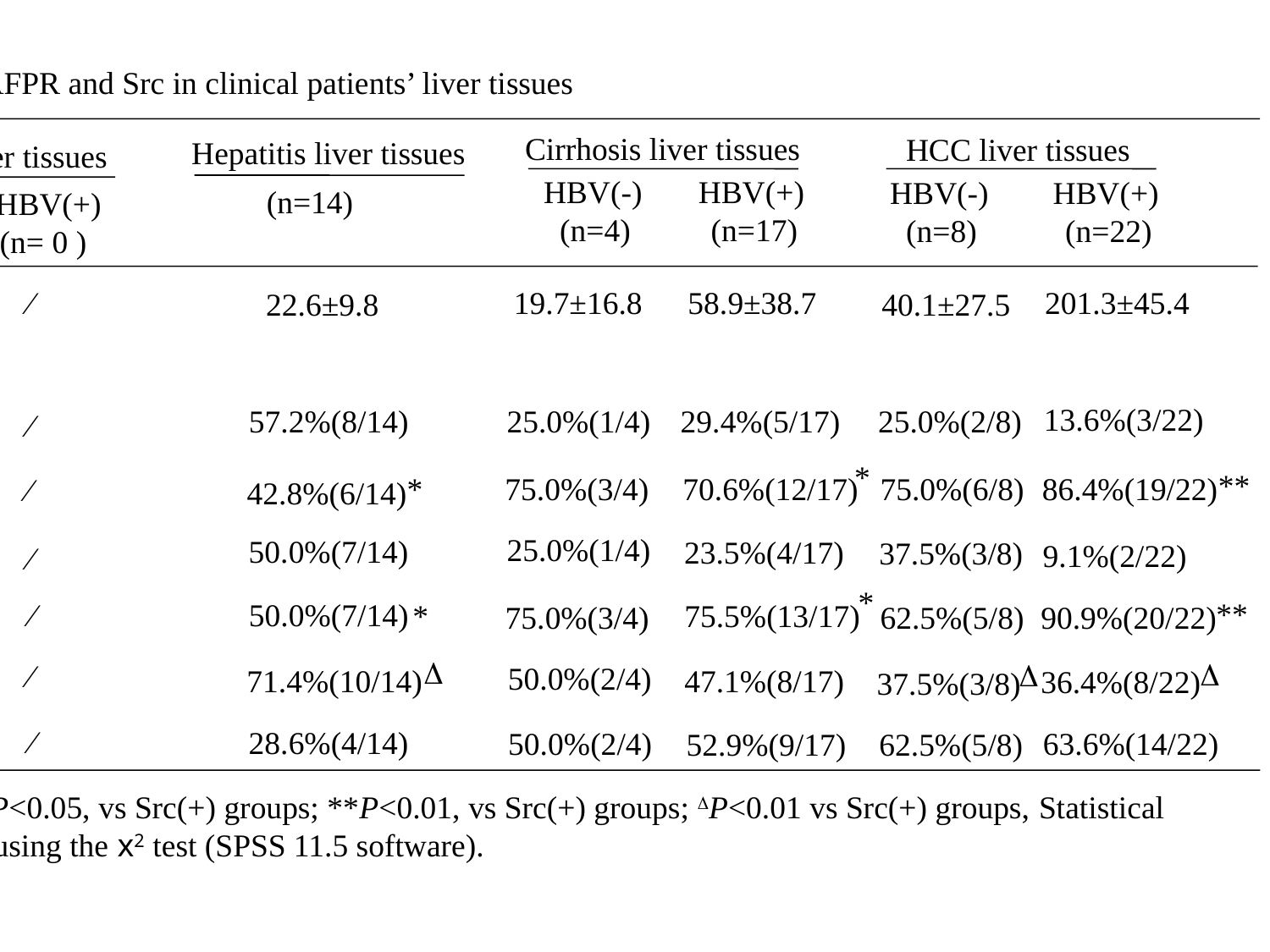

Table 1. Expression of AFP, AFPR and Src in clinical patients’ liver tissues
Cirrhosis liver tissues
HCC liver tissues
Hepatitis liver tissues
Normal liver tissues
HBV(-) HBV(+)
 (n=4) (n=17)
HBV(-) HBV(+)
 (n=8) (n=22)
(n=14)
HBV(-) HBV(+)
(n= 6 ) (n= 0 )
Analyzed items
Serum AFP
 (ng/ml)
8.5±4.6
58.9±38.7

19.7±16.8
201.3±45.4
40.1±27.5
22.6±9.8
IH stained
13.6%(3/22)
57.2%(8/14)
25.0%(1/4)
29.4%(5/17)
25.0%(2/8)
100%(6/6)

(-)
AFP
(+)
*
**
0%(0/6)
75.0%(3/4)
70.6%(12/17)
75.0%(6/8)
86.4%(19/22)
*

42.8%(6/14)
(-)
AFPR
(+)
25.0%(1/4)
50.0%(7/14)
23.5%(4/17)
37.5%(3/8)
100%(6/6)
9.1%(2/22)

*
0%(0/6)
**

50.0%(7/14)
*
75.5%(13/17)
75.0%(3/4)
90.9%(20/22)
62.5%(5/8)
(-)
Src
(+)
100%(6/6)

50.0%(2/4)
71.4%(10/14)
47.1%(8/17)
36.4%(8/22)
37.5%(3/8)
0%(0/6)

28.6%(4/14)
50.0%(2/4)
63.6%(14/22)
62.5%(5/8)
52.9%(9/17)
Note:  represent non data; *P<0.05, vs Src(+) groups; **P<0.01, vs Src(+) groups; ΔP<0.01 vs Src(+) groups, Statistical significance was determined using the x2 test (SPSS 11.5 software).
Δ
Δ
Δ
